# Supplementary material for: Are carbon emissions trading and green financial instruments synergistic? -Comprehensive quantitative research based on content analysis
Source: PLoS One. 2024 Mar 7;19(3):e0298601. doi: 10.1371/journal.pone.0298601 (PMC10919601; doi:10.1371/journal.pone.0298601)
Supplement: S1 Data — (ZIP) [file pone.0298601.s001.zip › Data/地级市Z_F工作报告.zip!/╕╩╦α/╬Σ═■╩╨/└┤╫╘╣1⁄2╓┌║┼í╛┬φ┐╦╩2╛▌═°í┐_2019.docx]

各位代表：

　　我代表市人民政府，向大会报告政府工作，请予审议，并请市政协委员和列席人员提出意见。

2018年工作回顾

　　过去的一年，是全市发展中负重爬坡、攻坚克难、扭转被动局面的一年，更是保持定力、夯实基础、开启新征程的一年。在省委、省政府和市委的坚强领导下，我们认真学习贯彻习近平新时代中国特色社会主义思想和党的十九大及十九届二中、三中全会精神，深入贯彻落实习近平总书记视察甘肃重要讲话和“八个着力”重要指示精神，按照高质量发展要求，突出抓好脱贫攻坚和生态环保两大基础性底线性任务，大力发展绿色生态产业，持续保障和改善民生，全市经济社会发展取得新的成效。

　　——经济运行回升向好，实现了质量和效益的同步提高。主要经济指标从三季度起全面回升，全年呈现“五升五稳”特点：实现生产总值469.27亿元，由2017年的下降2.8%回升到增长4.8%；第二产业增加值131.72亿元，由下降17.3%回升到增长3%；工业增加值66.38亿元，由下降27.3%回升到增长4%；规上工业增加值43.78亿元，由下降36.2%回升到增长5.7%；一般公共预算收入30.45亿元，由下降4.4%回升到增长7.1%。实现农业增加值123.22亿元，增长6.5%；第三产业增加值217.01亿元，增长5.2%；社会消费品零售总额205.72亿元，增长7.3%；城镇居民人均可支配收入27668元，增长8.2％；农村居民人均可支配收入11518元，增长8.7％，均保持了稳定增长态势。

　　——脱贫攻坚取得阶段性成果，夯实了全面建成小康社会的基础。全市退出贫困村73个，减少贫困人口2.95万人，贫困发生率由2017年的3.86%下降到1.81%。凉州区、民勤县通过省级评估验收摘帽退出。大抓产业扶贫，着重打造沿山、沿川、沿沙三大产业带，突出发展牛羊鸡菜果菌薯药八大特色产业，顶牛肉牛、中天羊业、天沪香菇、天禾菌业等一批产业项目带贫益贫作用日益凸显。天祝县获誉“中国高原藜麦之都”。古浪县黄花滩移民区万只种羊繁育基地被确定为甘肃省优质种羊繁育基地。

　　——生态人文家园建设成效明显，增强了群众的获得感和幸福感。大力弘扬“崇文尚德、包容创新”的武威城市精神及“勤朴坚韧、众志成城、筑牢屏障、永保绿洲”的民勤防沙治沙精神。八步沙“六老汉”三代人治沙造林先进事迹入选“伟大的变革——庆祝改革开放四十周年大型展览”，受到省委、省政府表彰。中心城区正阳路绿地公园、民勤东湖体育公园和人民公园、古浪阳哇山休闲广场、天祝和谐公园建成开放。新增城区绿地206.7万平方米，比2017年翻了一番多。公交公营改革全面完成，建成智慧、绿色公交体系。天马剧院投入使用，举办“新春文艺演出季”系列活动，丰富了群众精神文化生活。我市荣获创建全国文明城市提名城市，被新华网评为“最美中国·优质旅游目的地城市”和“首批全国文化旅游胜地” ，被命名为“甘肃园林城市”。凉州区被确定为省级全域旅游示范区创建单位，民勤县被命名为“甘肃园林县城”。

　　——重大带动性项目进展顺利，培育了转型升级发展的新动能。兰州至张掖三四线铁路中川机场至武威段获批启动开工。武威民用机场选址报告获国家民航局批复。我国首台自主知识产权医用重离子治疗系统临床试验治疗圆满完成。全省最大的文化旅游综合体PPP项目——武威雷台文化旅游综合体开工建设。甘肃（武威）国际陆港进境肉类指定查验场启动运营。伊利集团日加工1800吨液态奶、海升集团智能温室及水果胡萝卜种植加工、牧原股份100万头生猪养殖加工等一批产业项目落地，为高质量发展增添了新的动能。

　　——绿色发展走深走实，顺应了高质量发展的时代要求。扎实推动全域生态文明建设，编制实施县域经济、绿色生态产业、全域旅游发展规划，与中国农业科学院签订战略合作协议，制定构建生态产业体系推动绿色发展崛起实施方案，出台支持文化旅游产业发展的“黄金21条”政策措施。全市生态产业增加值达到133.65亿元、列全省第4位，占全市GDP的28.48%、列全省第2位。

　　全市经济社会发展实践充分证明，“走生态优先、绿色发展之路，努力建设经济强市、生态大市、文化旅游名市，全力打造生态美、产业优、百姓富的和谐武威”发展思路，全面贯彻了党的十九大精神，符合武威市情实际，顺应发展大势和群众愿望，必将引领我市步入高质量发展的快车道。

　　一年来，主要抓了八个方面的工作：

　　（一）以敢死拼命精神打好脱贫攻坚战。聚焦“一超过两不愁三保障”目标，制定打赢脱贫攻坚战三年行动实施意见，压实各级责任，完善“一户一策” 精准脱贫计划，到户到人精准落实脱贫措施。市、县区整合涉农资金11.84亿元，财政投入专项扶贫资金12.52亿元、增长52.1%；发挥财政资金撬动作用，落实特色产业发展工程贷款30亿元。下大力气解决贫困群众稳定增收问题，出台支持扶贫龙头企业、农民专业合作社、扶贫车间等发展的政策措施，引进德青源金鸡、康美牛羊、达康牧业、嘉禾农业等龙头企业，实现了龙头企业、农民专业合作社、特色产业对所有贫困村和有产业发展需求的贫困户全覆盖。贫困村发展特色种植21.91万亩，新建戈壁农业5996亩；建成光伏扶贫项目28.79兆瓦，惠及贫困户4818户；建立扶贫车间66个，吸纳贫困人口就业1086人。易地搬迁贫困人口1760人。新建续建贫困地区集中供水工程24处。新建改建贫困村幼儿园14所、中小学15所、标准化卫生室47所。贫困地区学前三年毛入园率、义务教育巩固率分别达到89.18%、99.91%。全面实施“先诊疗后付费”、“一站式结报”等政策，贫困人口合规医疗费用补偿比例提高到85%。深化东西部扶贫协作和中央单位定点扶贫，落实帮扶资金7130万元，实施了一批产业协作、农产品销售、劳务培训输转等项目。

　　统筹推进脱贫攻坚与乡村振兴。农村“三变”改革全面推开，实现农户和村集体分红4333万元。争取城乡建设用地增减挂钩跨省统筹调剂指标1370亩，交易金额4.11亿元，为脱贫攻坚和乡村振兴拓宽了资金来源。建成农村公路2137.6公里。持续开展全域无垃圾专项行动，推进农村“四化六有”改造，创建“千村美丽”示范村40个、“万村整洁”村150个。整治高价彩礼等突出问题，探索精神扶贫“文明股”分红奖励，培育乡风文明新风尚。

　　（二）深入推进全域生态文明建设。坚决落实生态文明建设责任，全面深入开展祁连山生态环境问题整改整治“回头看”，成立重点难点问题专责组和29个专责小组，逐项现场勘查论证，完善整改整治方案。至目前，除少数因季节性因素影响生态恢复的问题外，其它生态环境问题和“绿盾2018”专项巡查指出的神树水电站9个问题均完成整改。山水林田湖草生态保护修复工程累计完成投资23.89亿元。祁连山国家公园体制试点完成区划勘界和自然资源本底调查。实施国土绿化倍增行动，完成人工造林51万亩，封山（沙）育林（草）45.62万亩。“一带一路”生态治理民间合作国际论坛在我市成功举办。凉州九墩滩国家沙漠公园获批。编制报批《民勤生态建设示范区规划》。巩固提升石羊河流域重点治理成效，民勤蔡旗断面过水量3.85亿立方米，较约束性目标多9500万立方米，民勤盆地地下水开采量控制在约束性目标以内。污染防治攻坚战取得明显成效，凉州城区优良天气率达到93%，大气质量持续改善。全面落实市县乡村四级河长制、湖长制，县级及以上城市集中式饮用水水源地水质全部达标。全面开展乡镇、村规划建设管理突出问题清理整治，开展废旧宅基地复垦2.46万亩，拆除违规设置的广告牌10463面，拆违拆临215.75万平方米、复垦复绿13.46万平方米，清理“五未”土地及违规配置土地7.57万亩、处置5.54万亩，清理整治“大棚房”问题122个410.62亩，为全市高质量发展腾出了空间、廓清了障碍。

　　（三）着力推动产业结构转型升级。现代农业发展水平稳步提高，发展蔬菜、中药材等特色优势作物193万亩，推广高效农田节水技术257.2万亩、旱作农业50.7万亩，规模化养殖比重达到74%。累计认证“三品一标”农产品226个，建成出口农产品基地82.65万亩。培育农业产业化龙头企业147家、家庭农场490家、农民专业合作社9412家。生态工业焕发新的生机，坚持改造提升传统产业和培育发展新兴产业并重，实施工业项目108项，达利、青啤、正阳等“农字号”企业新线建成、产能扩大，众兴菌业、宏氟锂业等项目建成投产。全市新能源装机达到303万千瓦，清洁能源发电量占到总发电量的63.6%。加强工业经济运行调度服务，“一企一策”推进复产增产，为101户小微企业发放互助担保贷款7.29亿元。文化旅游产业势旺劲足，实施文旅项目84项，完成投资30.5亿元。武威历史文化街区保护建设项目正式签约，苏武沙漠大景区摘星小镇、沙漠戈壁农业示范园等一批项目加快建设，乌鞘岭国际滑雪场（二期）、沙漠雕塑国际创作营建成运营，普康田园综合体、凉州蜻蜓村、民勤红旗谷、古浪金水源、天祝大红沟等乡村旅游示范村加快培育，凉州植物园、西郊公园、湿地公园等加紧建设。中国社科院历史所凉州文化研究基地成立。举办第二届凉州文化论坛暨中国·河西走廊第八届有机葡萄美酒节、凉州文化与丝绸之路国际学术研讨会、“凉州讲坛”、“凉州文化沙龙”，凉州文化品牌影响力不断扩大。“天马行空·自在武威”文化旅游形象主题宣传语被列入“2018年旅游业最美中国榜”。全年旅游接待人数1513.8万人次，实现旅游综合收入84.4亿元，分别增长27.8%、33.3%。推进甘肃（武威）国际陆港基础建设及进出口贸易实体化运营，开通运行中俄木材回程班列、国际陆海贸易新通道回程班列，成功承办第四届中俄木材贸易投资大会。红星时代广场、万达广场、士林不夜城等商业综合体建成运营，带动了第三产业和城市经济升级发展。

　　（四）强力推进项目建设。坚持招大引强、精准招商，实行重大项目“五个一”包抓责任制，开展项目建设攻坚行动，中科院钍基熔盐堆核能系统实验平台配套设施、凉州城区第二水源地等项目建设进展顺利。北仙高速民勤至武威段及凉州绕城连接线建成118公里，G312线古浪金三角至凉州丰乐段改建项目建成，凉州至古浪、大靖至干城、炭山岭至赛什斯等一批普通省道和旅游公路加快建设。红崖山水库加高扩建工程全面建成并蓄水运行，古浪黄花滩移民区220万方调蓄水池开工建设。组织开展“春晖行动在武威·我与家乡共发展”系列恳谈活动，实施招商引资项目360项，到位资金212.58亿元。全年实施500万元及以上项目638项，44项省市列重大项目完成投资166.5亿元，有力促进了投资回升向好。

　　（五）扎实创建全国文明城市。制定实施创建全国文明城市三年规划，启动“三项革命”、“三项行动”，发布市民文明公约、文明行为规范，从烟头不落地、礼让斑马线等具体行为抓起，加大交通秩序、环境卫生等方面整治力度，城市文明形象和市民文明素质明显提升。编制完成中心城区13个片区控制性详细规划和城市风貌、色彩、交通系统等9个专项规划，首次实现城市总体规划、控制性详细规划、专项规划全覆盖，为提高城市工作的全局性、城市建设的系统性、城市发展的宜居性奠定了坚实基础，规划引领和约束作用进一步凸显。着力补齐民生领域短板弱项，新建改建城市（县城）公厕110座、公共停车场22个，打通城区“断头路”16条，治理背街小巷81条，改造老旧小区23个，整修检查井井盖4125个。新建智慧公交候车亭328个，开通夜班专线和学生晚自习专线，办理免费公交卡11.8万张。城乡环境面貌和公共服务功能持续改善，群众的幸福感、自豪感明显提升。

　　（六）紧盯难点堵点痛点深化改革。政府机构改革顺利实施。国资国企改革和工业园区管理体制改革扎实推进。认真落实“三去一降一补”改革举措，为企业降低用电成本7043.2万元。在全省率先推行政务服务“一窗受理、集成服务”改革，公布“最多跑一次”事项964项，取消各类证明事项364项。企业开办时间压缩到5天，新增市场主体1.94万户。深化知识产权领域改革，申请专利2066件，新认定国家级知识产权示范企业1家、优势企业2家。着力防范化解政府性债务风险，化解存量债务71.6亿元。帮助企业处置不良贷款29.6亿元。税收征管、国有林场等改革任务全面完成，供销社综合改革、户籍制度改革稳步推进。

　　（七）全力保障和改善民生。一般公共预算支出迈上200亿元新台阶，用于民生支出占一般公共预算支出的84.3%。新增政府债券和外债转贷资金23.53亿元，支持公益性项目、土地储备和棚户区改造。创新财政投入方式，多渠道筹措资金，多措并举促进民生改善。恢复了事业单位招考，公开招聘事业单位工作人员1022名。安置1013名城镇就业困难人员，城镇新增就业24846人，城镇登记失业率保持在3.07%的较低水平。居家养老服务改革全面推开，凉州区为近4万名老年人提供居家养老服务，三县居家养老信息平台全面运行。社会保障参保人数持续增加，保障水平稳步提高。城乡低保和特困供养提标工作全面完成。新建改建中小学103所、村幼儿园21所，消除城区中小学“大班额”193个，古浪县通过义务教育发展基本均衡县国家评估认定。凉州医院异地迁建、民勤县医院门急诊医技综合大楼加快建设，我市荣获“全国基层中医药工作先进单位”。完善城乡居民基本医疗保障政策，开通异地就医直接结算和养老保险关系转移直办平台。棚户区改造项目开工20148套（户），基本建成17648套（户）。改造提升“乡村舞台” 83个，建成乡镇（社区）体育健身中心10个、村级体育场110个，实现村级健身场所全覆盖。建成“放心粮店”12个、应急粮食供应网点135个。凉州城区新增天然气入户2.13万户。安全生产形势稳定好转，群众饮食用药安全有效保障。推进社会矛盾和重点信访问题集中排查化解，重点信项妥善解决。严格落实治欠保支“四项制度”，解决拖欠农民工工资问题。我市被评为“2018年人民网网民留言办理先进单位”。“智慧武威·雪亮工程”有序推进，基层网格化管理全面落实。扫黑除恶专项斗争取得阶段性战果，打掉黑恶势力犯罪团伙及村霸17个。平安武威建设成效显著，群众安全感综合测评位居全省第一。全市民族团结、宗教和顺，社会大局和谐稳定。

　　（八）从严从实加强政府自身建设。树牢“四个意识”，坚定“四个自信”，坚决做到“两个维护”。坚决肃清火荣贵流毒和影响，切实净化政治生态，努力构建清清爽爽的同志关系、规规矩矩的上下级关系和干干净净的政商关系。广泛深入开展宪法学习宣传和“七五”普法活动，全社会尊法学法守法用法氛围日趋浓厚。修订《市政府工作规则》，实行市政府常务会议“学法一刻”制度，严格落实项目建设“三性”论证和重大行政决策合法性审查、风险评估、专家咨询机制，全面推行法律顾问和公职律师制度。自觉接受市人大及其常委会法律监督、工作监督和市政协民主监督，自觉接受社会舆论和人民群众监督。64件人大代表意见建议、158件政协委员提案全部办结。审计监督作用不断强化。严格落实中央八项规定及其实施细则精神和省委、市委实施办法，深入开展“转变作风改善发展环境建设年”、“深化放管服改革工作突破年”和政府系统“十不准三基本”研判整改专项活动，党风廉政建设和反腐败斗争取得积极成效。

　　同时，统计调查、气象服务、防震减灾、人防、消防安全、公共资源交易、机关事务、双拥、工会、共青团等工作扎实推进，妇女儿童、老年人和残疾人权益得到有效保障，邮政、档案、地方志、保密、外事、侨务、住房公积金和慈善事业等工作取得新成绩。

　　各位代表，2018年，我们走得很艰辛，也很坚实。面对全市政治生态、自然生态、经济生态过去遭到严重破坏，经济下行等各种困难问题叠加碰头的局面，取得这样的成绩实属不易。这是省委、省政府和市委正确领导的结果，是市人大、市政协和社会各界监督支持的结果，是全市人民共同努力的结果。我代表市人民政府，向全市各族人民，向各民主党派、工商联、无党派人士、人民团体，向离退休老同志和社会各界人士，向驻武人民解放军、武警官兵、中央和省属单位，向民营企业家、投资者，向支持我市脱贫攻坚的天津市蓟州区、交通银行、中国化工集团和省直有关部门单位，向所有关心支持武威改革发展稳定的同志们、朋友们，表示由衷的敬意和感谢！

　　各位代表，思危方能居安，知难才能求进。面对新时代新要求新任务，我们清醒认识到，全市经济社会发展还存在许多亟待解决的困难问题。一是高质量发展的势头还不够强劲，新动能支撑作用还未完全显现，一些生态产业项目还在谋划、建设阶段，民间投资的潜力尚未充分挖掘和释放，结构调整、发展转型正在克难攻坚，促进固定资产投资、招商引资稳定增长的压力依然很大。二是剩余贫困人口脱贫难度大，产业增收基础还不牢靠，专业合作社、龙头企业与贫困群众的利益联结还不紧密，带贫作用亟待增强。三是中心城区、县城及重点集镇、工业园区环保基础设施建设仍是短板，大气、水、土壤污染防治措施落实还不完全到位，生态文明建设任务重、时限紧。四是城区停车难、老旧小区物业管理弱、城区学校条件差、“大班额”等问题尚未完全解决，一些乡镇卫生院和村卫生室基础设施不完善、医疗设备落后、专业人才缺乏，居民健康服务保障能力亟需加强。五是一些干部作风不严不实，不担当、不作为、不会为，以说代做、靠转替干，工作不落实、落不实的问题依然存在。对此，我们将坚持问题导向、效果导向，以更加有效的措施、更加务实的作风，努力把各项工作做实做好。

2019年目标任务和重点工作

　　今年是新中国成立70周年，是西部大开发战略实施20周年，是为全面建成小康社会打下决定性基础的关键一年。我们必须坚定发展信心，保持发展定力，蹄疾步稳地推动全市高质量发展。

　　今年政府工作的总体要求是：以习近平新时代中国特色社会主义思想为指导，全面贯彻党的十九大和十九届二中、三中全会精神，统筹推进“五位一体”总体布局，协调推进“四个全面”战略布局，深入落实习近平总书记视察甘肃重要讲话和“八个着力”重要指示精神，坚持稳中求进工作总基调，坚持新发展理念，坚持以供给侧结构性改革为主线，贯彻“巩固、增强、提升、畅通”方针，按照省委十三届七次全会暨省委经济工作会议、市委四届八次全会暨市委经济工作会议的部署要求，坚定不移走生态优先、绿色发展之路，继续打好三大攻坚战，统筹推进稳增长、促改革、调结构、惠民生、防风险工作，进一步稳就业、稳金融、稳投资、稳预期，奋力开创高质量发展新局面，为建设经济强市、生态大市、文化旅游名市，打造生态美、产业优、百姓富的和谐武威打下坚实基础，以优异成绩迎接新中国成立70周年。

　　经济社会发展的主要预期目标是：生产总值增长5%以上，固定资产投资增长6%左右，一般公共预算收入增长2%以上，社会消费品零售总额增长8%，城乡居民人均可支配收入分别增长7.5%、8.5%，居民消费价格指数涨幅控制在3%以内，单位生产总值能耗、能源消费总量和主要污染物排放量控制在省上下达的目标以内。

　　重点抓好八个方面的工作：

　　（一）紧盯目标加油冲刺，确保打赢脱贫攻坚战。

　　聚焦深度贫困地区和特殊贫困人口，坚持质量标准，夯实精准帮扶、产业脱贫、各方责任、基层队伍和工作作风基础，一鼓作气、决战决胜，确保天祝县、古浪县摘帽退出，全市减贫2.29万人以上，退出贫困村120个。

　　突出抓好扶贫产业。着力在沿山、沿川、沿沙三大产业带布局、落地一批牛羊鸡菜果菌薯药八大特色产业项目，加快推进德青源金鸡产业、浪美牛羊精深加工及鲜食面生产、南阳山藜麦产业园、食用菌产业园等建设，完善带贫益贫机制，带动扶贫特色产业发展。加大精准扶贫专项贷款、特色产业发展工程贷款、农产品收购贷款投放力度，积极争取产业发展投资基金，实现贫困村综合金融服务室全覆盖，全面推行特色农业保险政策，保障贫困群众稳定增收。持续引进和培育能够带动贫困户发展的产业组织，抓好农民专业合作社规范提升，做好要素保障、技术服务、产品销售和品牌创建等工作。大力发展光伏扶贫、扶贫车间、电商扶贫等新业态，新建扶贫车间80家以上，引导机关和国有企事业单位干部职工带头参与消费扶贫。

　　全力补齐短板弱项。强化易地扶贫搬迁后续产业培育，统筹做好基础设施建设、公共服务配套、就业增收、拆旧复垦等工作。深化农村住　　房安全大排查“回头看”，按时保质保量完成古浪县、天祝县危房改造。全力推进黄花滩生态移民暨扶贫开发调蓄供水工程，解决移民区用水短缺问题。实施农村饮水安全巩固提升工程16项，着力提高农村水质达标率和供水保证率。落实控辍保学、贫困生免补和生源地贷款等政策，确保义务教育巩固率保持在99.9%以上。落实健康扶贫各项政策，做好大病专项救治和慢病管理工作。研究解决收入水平略高于建档立卡贫困户的群体缺乏政策支持等问题，在产业、就业等方面有针对性地帮扶。坚持扶贫同扶志、扶智相结合，运用“群众知情会”、“学生家长宣讲会”等行之有效的方式，提振贫困群众的精气神，培训贫困劳动力1.2万人以上，激发脱贫内生动力。加强产业项目、人才技术、劳务输转等方面合作，提高东西部扶贫协作和定点帮扶工作实效。更好发挥驻村帮扶工作队和帮扶干部的作用，坚决有力彻底整改中央脱贫攻坚专项巡视、国家和省级考核、巡查、审计反馈的扶贫领域问题。

　　推进乡村振兴与脱贫攻坚有机衔接。坚持把乡村振兴作为新时代“三农”工作总抓手，强化规划引领，实现乡镇、村规划全覆盖。做大做强乡村特色产业、“五小”产业，促进产业融合发展，让农民在生产、加工、流通等各环节得到更多增值收益。推进高标准农田建设，强化农业科技集成创新，确保全市粮食总产量稳定在100万吨以上。统筹抓好村镇水、电、路等基础设施和教育、卫生、文化等公共服务设施配套建设。学习浙江“千万工程”经验，全面推开以农村垃圾污水治理、厕所革命和村容村貌提升为重点的农村人居环境整治，建设一批农村生活垃圾处理站和污水处理站，统筹做好农户改厕技术指导、工程监管、粪污处理、后期维护等工作，真正把这一民生实事办实办好。推动全市三分之一以上的乡镇完成“四化六有”改造，实现“千村美丽”示范村、“万村整洁”村数量增长、质量提升。发挥群众自治组织和村规民约作用，持续治理高价彩礼，推进殡葬改革，破除薄养厚葬陋习，倡导文明新风。

　　（二）坚决打好污染防治攻坚战，筑牢生态安全屏障。

　　深入贯彻习近平生态文明思想，将良好生态环境当作最普惠的民生福祉，用最严格制度、最严密法治促进生态环境持续改善。

　　全面整改各类生态环境问题。按时限、高质量完成祁连山生态环境问题、中央和省级环保督察及自然资源资产离任审计反馈问题的整改，严格整改验收标准，确保整改成效经得起实践和历史的检验。严格落实《祁连山国家级自然保护区管理条例》，积极开展祁连山国家公园体制试点工作，持续推进山水林田湖草生态保护修复工程。

　　打好蓝天碧水净土保卫战。全面推行煤改气、煤改电、改炕、改灶和清洁煤替代工程，完成柴油车治理8000辆，所有燃煤锅炉全部实现达标排放并建设封闭的堆煤（渣）场。强化工业园区、企业“三废”治理，坚决消除各类污染。严格落实河长制、湖长制，全面完成县城（工业园区）垃圾、污水、污泥处理工程建设。加大城市黑臭水体专项整治力度，开展县级以上集中式水源地环境保护专项行动，严格保护水体和饮用水水源，确保水质稳定达标。完成农用地土壤污染状况详查，抓好农业面源污染防治，确保土壤环境安全。

　　推进国土绿化递增行动。认真实施祁连山生态恢复、防沙治沙、绿洲绿化美化、湿地保护恢复、林业产业培育五大工程，推进城镇绿化、村庄绿化、景区（点）绿化、产业园区绿化、通道绿化、农田林网建设、林业有害生物防治、林业科技创新八大行动。坚持科学防沙治沙，争取《民勤生态建设示范区规划》列入国家专项规划，推进防沙治沙同发展沙漠生态经济、生态旅游和沙产业有机结合。弘扬践行八步沙“六老汉”三代人治沙造林和民勤防沙治沙精神，推动全域生态文明建设向纵深发展。完成营造林80万亩，其中人工造林36万亩、封育38万亩、提质改造6万亩。

　　实施国家节水行动。推进农业节水、工业节水、城镇节水、水权水价和水管体制改革、水资源精细化管理、完善制度体系六大节水行动，实施高效节水灌溉、农业用水计量设施和城镇供水管网更新改造、工业节水工艺改造等项目，推广农田高效节水技术250万亩、旱作农业45万亩。持续推进水价改革，健全完善有利于促进节约用水和生态补偿的水价体系。优化水资源配置，促进区域水资源平衡，倒逼产业结构、种植结构调整，促成全社会节水浓厚氛围，建设国家节水行动示范区。

　　（三）着力发展生态产业，打造高质量发展新引擎。

　　全面落实十大生态产业发展政策措施，全力争取省级绿色生态产业发展基金，加快实施重大带动性项目，扎实推动高质量发展。

　　提升发展现代农业。用工业化思维谋划现代农业，强化龙头企业、农民专业合作社、大户能人的引领带动作用，规划建设一批特色产业园，促进土地适度规模集约经营、壮大产业规模、提升农业发展水平，新建戈壁生态农业1.5万亩，发展中药材、高原有机蔬菜等特色优势作物200万亩，努力打造具有武威特色的现代丝路寒旱农业。优化现代农业示范园区布局，着力打造凉州区国家现代农业示范区、民勤县六大特色农业产业园、古浪县黄花滩万亩戈壁农业示范基地、天祝县金强川10万亩绿色高原蔬菜标准化生产基地等一批高标准现代农业产业示范园。开工建设伊利集团智慧乳业、海升集团智能温室及水果胡萝卜种植加工、牧原股份生猪繁育加工、康美集团白牦牛屠宰及精深加工等项目，加快嘉禾佳蔬菜种植及有机酱菜加工、达康牧业屠宰及深加工、正阳马铃薯主食化等项目建设。抓好葡萄种植、酿造、产品包装、副产物精深加工及葡萄酒庄文化旅游，提升葡萄酒全产业链发展水平。加大农业品牌建设力度，新认证“三品一标”农产品10个以上。深入实施粮改饲、循环农业发展专项行动，推动畜禽粪污、秸秆、废旧农膜、尾菜等农业废弃物资源化综合利用。开展农业技术巡回服务，促进良种繁育、疫病防控和标准化生产。

　　大力发展生态工业。推动传统产业转型升级，培育发展新能源、先进制造、数据信息、生物医药等绿色产业，为生态工业发展注入新动能。全力支持青岛啤酒、达利食品、众兴菌业、普安制药、石岛玻璃等重点企业增线扩能。加强工业运行调度服务，实施“一业一策、一企一策”，强化要素保障，抓好企业复产增产、战略性新兴产业和高新技术产业培育、规下转规上等重点工作。强化科技创新支撑，加快科技成果转化应用，培育高新技术企业6家以上，实施省级科技计划项目15项以上。落实以知识价值为导向的分配政策，充分释放科技人员创新创业的热情和活力。加快工业园区整合和管理体制改革，一个园区、一个规划、一套班子、一批企业，优化园区功能定位，明确主导产业，盘活存量土地，出清僵尸企业，提升产业配套水平。

　　加快发展文化旅游产业。深入实施全域旅游和乡村旅游发展规划，落实好“黄金21条”政策措施，促进旅游与文化、体育、科普、康养、节会等深度融合。加快建设凉州区省级全域旅游示范区。着力抓好雷台文化旅游综合体、武威历史文化街区保护建设、天梯山大景区、民勤苏武沙漠大景区、神州荒漠野生动物园、古浪战役纪念馆红色旅游景区、乌鞘岭大景区、普康田园综合体、游客集散中心及房车露营地等重点项目建设。大力发展乡村旅游，创建一批示范点和星级农家乐。完善旅游交通体系，加强景区基础设施配套建设，提升管理服务智能化水平，优化旅游服务环境。办好第三届凉州文化论坛、第二届沙漠雕塑国际创作营暨大地艺术节、全国“凉州词”创作大赛，常态化举办“凉州讲坛”、“凉州文化沙龙”，扩大“天马行空·自在武威”文化旅游品牌影响力，力争旅游接待人数增长25%，综合收入增长30%。

　　积极发展生态环保产业。围绕低碳节能、污染防治、资源综合利用、清洁生产、清洁能源等重点领域，谋划实施一批重点项目，筑牢绿色发展根基。重点实施海藏湖生态治理工程、杨家坝河及海藏河水系生态综合治理、凉州城区第二水源地、静脉产业园、锂电池新材料产业园等项目，做实生态环保产业。

　　着力发展通道物流产业。主动融入国家“一带一路”战略，加快陆港基础设施建设，完善口岸通关功能，提升进出口贸易实体化运营水平，确保进境木材检验检疫监管区通过国家验收。与天府商品交易所合作成立西北（武威）国际木材交易中心，运营好进口肉类指定查验场、保税物流中心和“天马号”班列，积极引进培育木材、肉类贸易加工产业。扩大特色农产品出口，争取开工建设铁路国际集装箱场站，推动开放型经济发展。加快实施市场体系完善提升工程，推进中心城区老旧市场升级改造、区域中心专业市场、农产品产地批发市场、集贸市场和城乡消费品市场建设，完善特色农产品冷链物流体系，形成物流通道、物流园区和专业市场有机衔接、相互贯通的大流通产业格局。

　　（四）强化投资消费双向拉动，有效增强发展后劲。

　　更好发挥投资的关键性作用和消费的基础性作用，千方百计扩大内需，为经济稳定增长提供有力支撑。

　　扎实推进项目建设。盯紧抓牢现代农业、生态工业、文化旅游产业、生态环保产业、民生及基础设施建设五大类221个高质量发展重点项目，做实做细项目前期工作，强化协调调度，加快落地进程，确保完成投资181亿元以上。抓好中科院钍基熔盐堆核能系统实验堆、实验平台及配套项目建设，确保储能、制氢等后续延伸产业落地。全力保障兰张铁路三四线中川机场至武威段项目建设，争取武威民用机场立项获批。建成北仙高速民勤县城至西渠段、大靖至干城、南营水库至旦马、高坝至祁连、S316线至白塔寺景区等公路，新建改建农村公路750公里。推进凉州区南营水库除险加固、民勤县红崖山水库至青土湖连通生态输水、天祝县金强川灌区水源保障等水利基础设施改造提升工程。重离子医院、武威职业学院直属附属医院改扩建等项目建成运营，“智慧武威·雪亮工程”、公共交通系统改造提升、凉州医院异地迁建、市妇幼保健计划生育服务中心、养老服务体系建设等项目完成阶段建设任务。

　　精准谋划储备重大项目。围绕绿色生态产业、脱贫攻坚、生态环保等重点领域，抢抓政策机遇，精准谋划储备一批大项目好项目。开拓融资渠道，创新融资方式，加大基础设施补短板力度，规范、争取PPP项目，促进民间资本参与基础建设。锲而不舍招大引强、选商选资，既注重外引外联，更注重内部挖潜，抓大不放小，招远不舍近，力争全年招商引资到位资金增长6%以上。

　　大力促进消费增长。顺应居民消费升级趋势，制定落实促进三产发展、消费增长的具体措施。加强限上企业协调服务，抓好个体转企业、限下转限上工作，支持限上企业发展壮大。引导鼓励商贸流通企业开展多种形式的消费促进活动，支持品牌零售企业、住宿餐饮企业向农村延伸。加强和改善市场监督管理，全面营造安全放心消费环境。

　　（五）深入推进创城工作，打造“德城”“文城”“绿城”“清城”。

　　以创建全国文明城市为统揽，科学规划、依规建设、精细管理，持续推进“三项革命”、“三项行动”，开展城市功能配套完善提升年、环境卫生整治巩固年、公园绿地建设达标年、老旧小区改造突破年、物业管理服务规范年“五个年”活动，不断改善群众生活环境，提升生活品质。

　　完善城市功能。加快实施凉州植物园和西郊公园改造提升、县城绿化提质改造工程，建设一批中心城区及县城文化广场、园林漫步空间、街巷休闲游园，新增城区绿地100万平方米以上，建成森林公园4个、生态小游园25个。新建改建一批市政道路，优化城区路网结构。加快中心城区及县城路标、灯具、分道栏、垃圾箱、候车亭、门头牌匾等公共设施的新建改造，提升城市形象和品位。

　　开展文明创建。加快智慧城市建设，建成运行数字化管理服务平台，推进城市精细化管理。持之以恒抓好“遵守红绿灯”、“礼让斑马线”、“烟头不落地”等专项整治，治理城市脏乱差死角。大力弘扬社会主义核心价值观，让践行文明公约成为市民的自觉行为，不断提升全社会文明水平。

　　推进拆临拆违。以零容忍态度强力拆除违法违章建筑，坚决消除存量、杜绝新增，切实维护城乡规划的权威性、严肃性。依法依规清理整治“大棚房”、“五未”土地，进一步加大乡镇、村规划建设管理突出问题整治力度，全面清理整治停建“烂尾”工程，综合施策、大小齐抓，推动各类问题得到全面彻底整改。

　　（六）全面深化改革，增强发展动力活力。

　　贯彻中央和省委各项改革部署，结合实际，积极探索，把群众关注的热点难点问题作为改革的重要事项，小处切口、点上发力，加大改革力度，释放改革红利。

　　持续推进供给侧结构性改革。坚决防止产能过剩行业新增产能。严格用地供应，加快消化非住宅商品房存量。全面落实减税降费政策，进一步清理规范涉企收费，继续实施电价、直购电交易等改革举措，推动企业降本增效。坚持底线思维，防范化解金融风险，密切关注地方银行流动性风险，牢牢守住不发生区域性金融风险底线。加强政府债务管理，持续压减“三公”经费，以改革的思路、创新的办法拓宽融资渠道，搞建设、促发展。加快推进市属国有企业改革重组，健全完善企业法人治理结构，提高市场化运营水平。

　　争创一流营商环境。按期完成市、县区政府机构改革任务。深化“放管服”改革，大力推进“四办”改革，进一步压减办事流程和环节，政务服务事项网上可办率达到90%以上。推进工程建设项目审批制度改革，持续开展“证照分离”改革和“减证便民”行动，畅通网上咨询投诉渠道，切实方便企业、群众办事。真心实招支持民营企业发展，全面落实省委、省政府支持中小微企业高质量发展58条政策措施，积极搭建企业人才、用工招聘平台，强化银政企对接，完善融资担保体系，切实解决企业用工、融资等发展难题，引导、支持企业聚焦实业、做精主业，放心投资、放手发展。

　　推进农村综合改革。全面完成农村承包地确权登记颁证工作，规范土地流转管理，引导经营权向特色农业、新型农业经营主体有序流转。推进农村宅基地制度改革，完成农房一体调查登记发证。加快农村集体资产清产核资、确权登记、折股量化等工作，激活农村要素资源，推进农村“三变”改革，发展壮大村集体经济，不断增加农民财产性收入。落实承包土地经营权担保融资政策，探索集体资产股权质押贷款办法，打通金融服务“三农”各个环节。挖掘国有林场资源优势，提升经营管理水平，提高国有资产运行效益。稳步推进供销社综合改革。建设坚强有力的农业农村综合执法队伍，推进依法护农、法治兴农。

　　（七）切实保障和改善民生，增进人民群众福祉。

　　坚持以人民为中心的发展思想，把更多人力物力财力投向最贴近群众的地方，让高质量发展的成果更好惠及群众。

　　加强就业和社会保障。稳就业、增收入，重点支持高校毕业生、农民工、退役军人等群体创业就业，大力发展“归雁经济”，鼓励外出务工人员返乡创业就业，实现城镇新增就业2.1万人，城镇登记失业率控制在3.5%以内。扎实做好社会救助兜底保障，着力推进城镇困难职工解困脱困工作，落实城乡居民基础养老金正常调整政策，进一步提高城乡低保和特困供养标准，关心关爱残疾人、优抚对象等群体。发挥公积金制度作用和优势，加大对新市民租房购房补贴力度，强化对农村群众建房的规划、许可服务，保障和改善城乡群众居住条件。

　　统筹推进社会事业发展。优先发展教育事业，实施教育现代化提升工程，深入推进城乡义务教育一体化改革发展。加快推进职业教育产教融合发展，加快发展特殊教育、继续教育，促进民办教育健康发展。深化新时代教师队伍建设改革，树立良好师德师风，提升教师专业素质能力。加快推进医药卫生体制改革，稳步推进公立医院现代化管理制度建设，加大全科医生培养力度，加强医联体和县级医院重点专科建设，推进分级诊疗和家庭医生签约服务。完成重离子医疗设备注册和管理重构，尽快运营。加大医疗保险基金监管力度。加快建设多层次、智能化养老服务体系，完善社区居家养老服务功能，推进医养融合发展。加强未成年人思想道德建设，做好困境儿童和农村留守儿童关爱保障工作。加快公共文化服务体系建设，实施文化惠民、文化遗产传承保护工程，启动农村（社区）“一站式”阅览服务室建设。实施全民健身计划，多点布局笼式足球场、多功能运动场，新增全民健身点20个。广泛开展群众文体活动，引进发展品牌赛事。

　　提升社会治理水平。严格落实安全生产责任体系，持续强化道路交通、危险化学品、非煤矿山、建筑工地、食品药品、特种设备和人员密集场所等重点领域安全监管。加强应急处突力量和装备建设，提升应急综合防范处置能力。落实维稳工作责任制，有效化解矛盾纠纷和信访积案，开展“无欠薪”城市创建活动。着力推进信用信息归集共享，加快社会信用体系建设。加强双拥模范城（县）创建工作，推进军民融合发展。做好退役军人服务保障，维护军人军属合法权益。纵深推进扫黑除恶专项斗争，严厉打击各类违法犯罪活动。深入开展民族团结进步创建工作，依法管理宗教事务，维护社会和谐稳定，天祝县力争创建全国民族团结示范县。

　　群众盼的就是我们要干的，群众身边的热点难点就是我们工作的重点，要坚持眼睛向下、脚步向下，及时了解群众所思所盼，解决一批群众最盼最急最忧最怨的问题，重点办好10件民生实事：1.减少城区学校“大班额”100个以上，新增城区公办幼儿园园位1000个以上。2.继续开展城区学校基础设施三年改造提升行动，全面推进武威一中提升扩建项目，引进优质高中教育资源。3.市博物馆、图书档案馆、科技馆、城市规划馆建成开放。4.新建标准化村卫生室25所。5.规划建设中心城区及县城停车场24个，新增停车位5000个以上。6.提升改造老旧小区12个，惠及居民2200户以上；加装老旧小区电梯30部；实施城镇棚户区改造4955套（户）。7.新建改建中心城区及县城达标公厕50座以上。8.完成中心城区天然气入户1万户以上，覆盖率达到90%以上。9.强力推进农村人居环境综合整治，安装村道路灯2600盏以上，修建垃圾中转站17座，基本建成农村生活垃圾收集转运处理体系。10.做实做强社区警务室，建成标准化社区警务室290个以上，打造老百姓家门口的派出所。

　　（八）忠诚履职尽责，建设人民满意的服务型政府。

　　新时代新征程要求政府工作必须有新起色新作为。我们将以政府机构改革为契机，进一步转变职能、提高效能，勤政廉洁、狠抓落实，努力做到信念过硬、政治过硬、责任过硬、能力过硬、作风过硬，不辜负全市人民的期望和重托。

　　加强政治建设。坚持用习近平新时代中国特色社会主义思想武装头脑、指导实践、推动工作。开展“不忘初心、牢记使命”主题教育，树牢“四个意识”，坚定“四个自信”，坚决做到“两个维护”。坚持知行合一，自觉把讲政治贯穿于政府工作各个方面，落实到具体工作中、体现到实际行动上。

　　坚持依法行政。贯彻落实“七五”普法规划和法治政府建设实施纲要，严格依照法定权限和程序履行职责，运用法治思维和法治方式推动工作。完善政府立法机制，做好城市社会生活噪声污染防治、大气污染防治、物业管理、历史文化名城保护等条例的立法工作。依法接受市人大及其常委会监督，自觉接受市政协民主监督，主动接受社会监督和舆论监督。严格规范执法行为，使权力在阳光下运行。

　　强化担当作为。适应高质量发展要求，深入研究、敏锐捕捉政策机遇，加强谋划、高效对接，提高政策转化落地能力，努力做到善学善谋、善作善成。抓落实、强执行，重诺守信，着力整治不落实、乱落实、假落实、少落实、慢落实、机械落实、应付式落实等形式主义、官僚主义问题，说了就算、定了就干、办就办好。坚持委任责成、守正出新，察实情、说实话、出实招、求实效。主动担责尽责，不讲干不成的理由，多想干成事的办法，用创新的思维、改革的办法、务实的举措破解发展难题，实事求是地化解历史遗留问题，推动各项工作提质提速提效。

　　建设廉洁政府。认真落实全面从严治党主体责任和“一岗双责”，严格执行中央八项规定及其实施细则精神和省委、市委实施办法，深入开展政府系统“十不准三基本”研判整改专项活动，做到履职尽责有原则、有规矩、有底线。健全权力运行制约和监督机制，依法加强审计监督，强化公共资源交易监管，持续整治群众身边的腐败和作风问题，把政府系统党风廉政建设和反腐败工作不断推向深入。

　　各位代表！

　　使命呼唤担当，奋斗成就未来。让我们更加紧密地团结在以习近平同志为核心的党中央周围，在省委、省政府和市委的坚强领导下，坚定信心，担当作为，务实苦干，扎实工作，为加快建设生态美、产业优、百姓富的和谐武威，决战决胜脱贫攻坚，全面建成小康社会而共同努力奋斗！
